# Nck2 promotes human melanoma cell proliferation, migration and invasion *in vitro *and primary melanoma-derived tumor growth *in vivo*

**DOI:** 10.1186/1471-2407-11-443

**Published:** 2011-10-12

**Authors:** Mélissa Labelle-Côté, Julie Dusseault, Salma Ismaïl, Aude Picard-Cloutier, Peter M Siegel, Louise Larose

**Affiliations:** 1 Programmes de biologie moléculaire, Faculté de Médecine, Université de Montréal, Montréal, Québec, Canada; 2 Department of Biochemistry and Goodman Cancer Research Centre, McGill University, Montreal, Quebec, Canada; 3 Polypeptide Laboratory, Division of Endocrinology, Department of Medicine and Research Institute of the McGill University Health Centre, McGill University, Montreal, Quebec, Canada

## Abstract

**Background:**

Nck1 and Nck2 adaptor proteins are involved in signaling pathways mediating proliferation, cytoskeleton organization and integrated stress response. Overexpression of Nck1 in fibroblasts has been shown to be oncogenic. Through the years this concept has been challenged and the consensus is now that overexpression of either Nck cooperates with strong oncogenes to transform cells. Therefore, variations in Nck expression levels in transformed cells could endorse cancer progression.

**Methods:**

Expression of Nck1 and Nck2 proteins in various cancer cell lines at different stages of progression were analyzed by western blots. We created human primary melanoma cell lines overexpressing GFP-Nck2 and investigated their ability to proliferate along with metastatic characteristics such as migration and invasion. By western blot analysis, we compared levels of proteins phosphorylated on tyrosine as well as cadherins and integrins in human melanoma cells overexpressing or not Nck2. Finally, in mice we assessed tumor growth rate of human melanoma cells expressing increasing levels of Nck2.

**Results:**

We found that expression of Nck2 is consistently increased in various metastatic cancer cell lines compared with primary counterparts. Particularly, we observed significant higher levels of Nck2 protein and mRNA, as opposed to no change in Nck1, in human metastatic melanoma cell lines compared with non-metastatic melanoma and normal melanocytes. We demonstrated the involvement of Nck2 in proliferation, migration and invasion in human melanoma cells. Moreover, we discovered that Nck2 overexpression in human primary melanoma cells correlates with higher levels of proteins phosphorylated on tyrosine residues, assembly of Nck2-dependent pY-proteins-containing molecular complexes and downregulation of cadherins and integrins. Importantly, we uncovered that injection of Nck2-overexpressing human primary melanoma cells into mice increases melanoma-derived tumor growth rate.

**Conclusions:**

Collectively, our data indicate that Nck2 effectively influences human melanoma phenotype progression. At the molecular level, we propose that Nck2 in human primary melanoma promotes the formation of molecular complexes regulating proliferation and actin cytoskeleton dynamics by modulating kinases or phosphatases activities that results in increased levels of proteins phosphorylated on tyrosine residues. This study provides new insights regarding cancer progression that could impact on the therapeutic strategies targeting cancer.

## Background

Melanoma skin cancer is one of the most devastating types of cancer, extremely aggressive with high metastatic potential. Melanoma metastasis to distant organs is the primary cause of human cancer-related deaths. Worldwide, the incidence of cutaneous malignant melanoma is increasing faster than any other type of cancer. Cutaneous melanoma originates from pigment-producing melanocytes localized at the epidermal-dermal junction in human skin and develops through different steps [1]. Among various hypotheses, it is proposed that these involve radial (RGP) and vertical (VGP) aberrant growth phases of preexisting nevi or at new site. Then to metastasize at distant sites, melanoma detach from a primary lesion, acquire motility and proteolytic activities to reach lymphatic and blood circulation and undergo growth to distinct organs, all this according to stepwise molecular changes involving defined genetic events [2,3]. However, the exact mechanisms underlying this devastating process are complex and somehow still poorly understood. From a molecular point of view, oncogenic activation of the mitogen-activated protein kinase (MAPK) pathway, due to somatic mutations in B-RAF (V600E), is frequently observed in melanoma (70%) [4].

In mammals, the family of Nck (non-catalytic region of tyrosine kinase) proteins is represented by two highly conserved members, Nck1 and Nck2, composed of three N-terminal SH3 (Src homology 3) domains followed by a unique C-terminal SH2 (Src homology 2) domain and devoid of any catalytic activity [5,6]. Like other SH2/SH3 domain-containing proteins, Nck1 and Nck2 behave as adaptor proteins by physically coupling activated membrane receptors to specific downstream effectors [7]. In mice, individual *Nck *knockout resulted in no phenotype, confirming redundancy of Nck proteins, while early embryonic lethality of the double *Nck *knockout mice revealed their crucial role in embryonic development [8]. However, regardless that Nck1 and Nck2 share high amino acid identity, and common cellular functions and binding partners, increasing evidence support specific roles and proteins interactions, as well as tissue expression patterns for these adaptors [7,9-15]. Previous studies have reported that overexpression of Nck1 in fibroblasts induces cellular transformation and that these cells form tumors in mice [16,17]. Furthermore, either Nck has been shown to cooperate with potent oncogenes (v-Abl and Ras) to transform cells, influence cell morphology and anchorage-independent growth [6]. Although, these studies strongly suggest a role for Nck in cancer development, the mechanism by which Nck oncogenic potential is achieved still remains to be established.

Originally the Nck1 cDNA was isolated from a human melanoma cDNA expression library using a monoclonal antibody produced against the human melanoma-associated antigen [5], which has no similarity with Nck1. This suggests that the Nck1 mRNA might be abundant in human melanoma. Most recently, the *Nck2 *gene was found as being overexpressed in human metastatic melanoma compared with non-metastatic melanoma lesions [18]. In agreement, the cancer microarray database Oncomine (https://www.oncomine.org/) reports *Nck2 *as a gene upregulated in several human cancer cell lines, including human melanoma. Therefore, the concept that deregulated expression of Nck adaptor proteins could contribute to promote melanoma development and/or progression deserves further investigation. In the present study, using human melanoma cell lines harboring the activating B-RAF (V600E) mutation, that are well defined for stage of cancer progression [19,20], we demonstrate that Nck2 protein and mRNA levels are increased in human metastatic melanoma cells compared with human primary melanoma cells that rarely metastasis. We show that Nck2 promotes cell proliferation, migration and invasion in human melanoma cells. In addition, using an *in vivo *xenograft model, we provide evidence that increased Nck2 expression in human primary melanoma cells promotes melanoma-derived tumor growth rate. Collectively, our findings indicate that Nck2 plays a role in human melanoma progression.

## Methods

### Cell lines

The Wistar melanoma cell lines (WM278, WM1232, WM115, 1205Lu, WM164, WM1617 and 451Lu) were obtained from Dr Meenhard Herlyn (PA, USA). Human Epidermal Melanocytes (HEM) cell line was purchased from Cell Applications Inc. Murine colon carcinoma cell (CT26, CT36 and CT51) were obtained from Dr. Nicole Beauchemin (McGill University, Montreal, QC). Breast cancer cell lines (MCF10, MCF7, T47D, MDA-MB-231) were kindly provided by Dr. Morag Park (McGill University, Montreal, Qc).

### Cell culture

Unless specified, all chemicals used in this study are from regular commercial sources. Cells were maintained at 37°C in 5% CO_2_-95%O_2 _atmosphere. HEK293, colon and breast cancer cell lines were grown in DMEM (Dulbecco's modified Eagle's medium) containing 10% FBS and supplemented with 100 Units/ml of penicillin, 100 μg/ml of streptomycin and 0.25 μg/ml of Amphotericin B. Melanoma cell lines were grown in RPMI 1640 supplemented with 2 mg/ml NaHCO_3 _and 0.3 mg/ml glutamine. MCF10 cells were grown in DMEM containing 5% Horse Serum (Invitrogen), 20 μg/ml of mouse epidermal growth factor (mEGF, Collaborative Biomedical Products), 10 μg/ml of insulin and 0.1 μg/ml of cholera toxin. To induce MCF10 cell differentiation, cells were grown in media in absence of mEGF but supplemented with 0.5 μg/ml of hydrocortisone for two days. HEM (human epidermal melanocyte) cells were grown in HEM media (Cell Applications Inc.) and cultured according to the manufacturer's instructions.

To analyze phospho-tyrosine proteins, cells were exposed to protein phosphotyrosine phosphatase inhibitor (pervanadate (Na_3_VO_4_) or bpVPhen, 100 μM, 15 min at 37°C) prior to be harvested and total cell lysates processed for anti-phosphotyrosine western blot as reported below. Alternatively, total cell lysates (2 mg protein) were incubated with indicated antibodies (4 μg) for 2 hours at 4°C and 40 μl of 50% slurry solution of Protein-A immobilized on Sepharose beads (Santa Cruz Biotech.) were added for an additional 2 hours of incubation at 4°C. Immunoprecipitated samples were washed 3X with lysis buffer before to be finally recovered in Laemmli buffer and processed for anti-phosphotyrosine western blot as reposted below.

### Antibodies

Nck polyclonal antibodies were raised by immunizing rabbits with GST-Nck fusion proteins as antigens. Crude serum samples were Protein-A-purified (ProChem, MA) and further tested for Nck specificity as described below. A pan-Nck antibody (1793), which recognizes both Nck isoforms was raised against residues 1-251 containing the three SH3 domains of human Nck1 as previously reported [21]. Nck1 antibody (2383) and Nck2 antibody (3313) were raised against isoform specific amino acid sequence in between the last SH3 and the SH2 domain of each Nck as reported earlier [15]. Other antibodies used are: CrkII (C-18), Integrin β3 (H-96), phospho-tyrosine (clone PY99), HA (Y-11) and GFP (B-2) from Santa Cruz Biotech. Antibodies against Integrin β1 (anti-CD29, clone 18), E-Cadherin (clone 34) and N-Cadherin (clone 32) were purchased from BD (ON, Canada). Antibodies to detect vinculin (clone h-VIN-1) and Tubulin (TUB2.1) were from Sigma-Aldrich, USA. Secondary antibodies coupled to HRP were from Bio-Rad Inc. Rhodamine-coupled to mouse anti-IgG was bought at Jackson ImmunoResearch Inc. Phalloidin-coupled to AlexaFluor^®^555 and 488 were purchased from Molecular Probes (Invitrogen, CA, USA)

### Cell lysis and western blots

Cell lysates were prepared in lysis buffer (50 mM HEPES pH 7.4, 150 mM NaCl, 10% Glycerol, 1% Triton X-100, 1.5 mM MgCl_2_, 1 mM EGTA, 10 mM Sodium Pyrophosphate, 100 mM Sodium Fluoride, supplemented with the protease inhibitors Aprotinin and Leupeptin at 1 μg/ml and PMSF at 1 mM. Lysates normalized for protein content (Bradford protein assay, Bio-Rad) were prepared in Laemmli buffer, heated, subjected to SDS-PAGE on 10% acrylamide gels and transferred onto nitrocellulose membranes. For western blot analyses, membranes were blocked in TBS (Tris-buffered saline) containing 10% dry milk and 0.1% Tween-20, and then incubated overnight at 4°C with indicated primary antibodies appropriately diluted in the blocking solution. For pY western blot, blocking and primary antibody solution was TBS containing 5% bovine serum albumin (BSA, Sigma) and 0.1% Tween-20. Next morning, the membranes were washed twice with TBS for 5 minutes followed by two 5 minutes washes using TBS-T (TBS-0.1% Tween-20) and two 5 minutes washes with TBS. The membranes were then incubated with secondary antibody appropriately diluted in milk-blocking solution for 1 hour and washed as above. Finally, signal was detected using ECL Plus Western Blotting Detection System (GE Healthcare, UK) and XR film exposure.

### RNA isolation and RT-PCR

Total RNA was isolated from melanoma cells using the TRIZOL (Invitrogen) according to the manufacturer's protocol. Briefly, cells from 100-mm dishes (1 × 10^6 ^cells) were suspended in 7.8 ml of TRIZOL. The aqueous and organic phases were separated after addition of chloroform. Precipitated RNA by isopropyl alcohol addition was washed in 70% ethanol and dissolved in RNase-free water. RNA concentration and purity (OD_260/280_) was measured using an Ultrospec 2100 Pro UV/visible Spectrophotometer (Fisher Scientific, ON). First-strand cDNA synthesis was generated by reverse transcriptase reaction in a final volume of 50 μl. For this, 2.0 μg of total RNA were mixed in a total reaction volume of 20 μl of RNAse free water containing 1 μM Oligo d(T)_20 _for Nck amplifications or 6 μg of Random Primers for 18S amplification. The reactions were incubated at 65°C for 5 min and quenched on ice. Then, the RT reaction was assembled by adding 10 μl of the 5X 1^st ^strand buffer (Invitrogen), 5 μl of 0.1 M DTT (Invitrogen), 2.5 μl of RNase Inhibitor (40 U/μl) (Invitrogen), 2.5 μl of 10 mM dNTPs, 5 μl of 50 mM MgSO_4 _and 2.5 μl of Superscript III (200 U/μl) (Invitrogen). Samples were incubated at 37°C for 50 min and deactivated at 70°C for 15 min. PCR amplification was performed using 0.5 μl of cDNA template in a final volume of 50 μl containing 5 μl of 10X PCR Enhancer buffer (Invitrogen), 1.5 mM MgCl_2_, 0.2 mM dNTPs, 50 pM of specific forward and reverse primers, 10 μl of Amplification buffer (Invitrogen) and 1 U of Taq DNA polymerase (Invitrogen) and DEPC water. Primers used were: Nck1 forward 5'-GCCAGATTCTGCATCTCCTG-3', Nck1 reverse 5'-ACACTTGCCCAGTATTTAGG-3', Nck2 forward 5'-CGAGTACCCCGCCAATGG-3' and Nck2 reverse 5'-CCCGTCACTGAGGACCACC-3'. Reactions were carried out on PTC-100 Programmable Thermal Controller (MJ Research Inc.) according to the following program conditions: initial denaturation at 94°C for 1 min, followed by 1 min at 94°C, 30 seconds of annealing (47°C for Nck 1, 53°C for Nck 2 and 55°C for 18S) and 1 min at 72°C. The final elongation step was 10 minutes at 72°C and the samples were kept at 4°C until analysis. PCR products were separated on a 1% agarose gels and imaged using an NIH Image J 1.30 system. Fifteen, 20, 25 and 30 cycles of PCR amplification products were analyzed to confirm that the amplification was in the linear range for each gene. Ratios of Nck1 and Nck2 over 18S were calculated from similar assays performed in triplicate and repeated three times.

### Cell transfection

Human HA-tagged Nck2 cDNA generously provided by Dr. Wei Li (University of Southern California, LA, CA) was subcloned into the retroviral vector pLXSN (Clonetech Laboratories Inc., CA, USA) and the viral particles produced using the GP2-293 cell line according to the manufacturers' instructions. Human Nck2 cDNA was also subcloned into the pEGFP-C1 plasmid (BD, NJ, USA). To establish stable clones of human WM278 primary melanoma overexpressing GFP or GFP-Nck2, cells plated in 100-mm dishes (1 × 10^6 ^cells) were transiently transfected with 10 μg of plasmid DNA (pEGFP or pEGFP-Nck2) using calcium phosphate and following selection with neomycin, clones were isolated, amplified and analyzed for GFP or GFP-Nck2 by western blot. 451Lu cells plated at 40-60% confluence were transfected with either 100 nM Nck2 or control siRNA 13379 (Ambion, Austin, TX) using Lipofectamine Plus reagent according to the manufacturer's protocol and analyzed for protein expression after 24 or 48 h.

### Proliferation assays

Briefly, cells (4 × 10^3^) were seeded in 96-well plates and 24, 48, 72 or 96 h after, cells were fixed by adding glutaraldehyde (20 min, final concentration 1%). Then, fixed cells were washed twice with deionised water and stained with Crystal Violet (20 min, 0.4% in 10% ethanol, Sigma). The excess of Crystal Violet was removed by washing the cells three times with water and finally, incorporated Crystal Violet was dissolved in 10% acetic acid and read at 570 nm using a spectrophotometer (Beckman Coulter). Wells without cells, but containing medium were used as blank value that was subtracted from all values. Data were expresses a raw OD at 570 nm or as ratio of OD at specific time point over initial OD at day 1.

### Wound healing assays

Cell migration was assessed in classical wound healing assays. Confluent monolayer cells in a 6-well plate were wounded using a plastic pipette tip (P200) and rinsed with PBS before to add back culture medium. The bottoms of the wells were marked to indicate where the initial pictures of the wound area were taken. After 8 h incubation at 37°C, pictures (10X) of the same areas were recorded using an Axiovert 200 M microscope (Zeiss) equipped with a CoolSnap™ES camera (Photometric^®^, Roper Scientific) and closure of the wound evaluated using Metamorph^® ^(V6.3, Molecular Devices Corp.).

### Cell invasion assays using Transwells

Melanoma cells (1 × 10^5^) resuspended in 10% serum containing medium were added to the top chamber of a Transwell (8 μm, DD Biosciences, NJ, USA) pre-coated with matrigel™ (BD Biosciences, NJ, USA) diluted in ice-cold PBS (175 μg/ml) at a total of 35 μg per well and allowed to migrate for 24 h. To evaluate the amount of cells that had invaded through each transwell, excess of media was discarded and the transwells were washed once with PBS and then placed in trypsin solution (0.025%) to release the invaded cells underside of the transwells and in the bottom chamber. Total invaded cells were estimated using Calcein AM (BD Biosciences, NJ, USA) as recommended by the manufacturer. Data were normalized according to the respective total amount of cells for each line plated at the same time in adjacent wells devoid of transwells to take into account variations in cell number between cell lines.

### Spheroid formation assays

Spheroid formation and culture in 3D were performed according to the hanging drop method [22]. Briefly, 2 × 10^4 ^cells in 20 μl of culture medium were suspended on the lid of tissue culture dishes containing 10 ml of culture medium for 48 h to form spheroids. Then spheroids were transferred in culture dishes containing culture medium and on 2% agar (Agar Select, Invitrogen, CA, USA) at the bottom. After 72 h of growth in suspension, individual spheroid has been transferred in 4-well plate containing 80% collagen type IV (PureCol^®^, Advanced BioMatrix) in RPMI without FBS. Following 30 min at 37°C to allow collagen polymerization, 500 μl of RPMI containing 10% FBS was added to each well. Images were recorded initially and at 12-24 hr intervals as reported above for wound healing assays. Spheroid invasion was determined qualitatively as either positive or negative comparing sequential images.

### Actin and focal adhesions

Cells were plated at 3 × 10^4 ^cells/well on glass coverslips pre-coated or not with various extracellular matrices and incubated in culture medium for 24 h. All steps were carried at room temperature and coverslips were rinsed with PBS between each step. Cells were fixed in freshly prepared 3.7% formaldehyde for 10 min, permeabilized in 0.2% Triton-X-100 for 5 min and blocked in 0.1% BSA for 30 min. For vinculin staining, cells were incubated with primary monoclonal anti-vinculin antibody (1:400) for 1 h and with a mixture of secondary tetramethylrhodamine isothiocyanate-conjugated phalloidin-conjugated goat mouse antibody (TRITC-GAM, Sigma) for 30 min. Actin staining was performed by incubating the coverslips for 30 min with Phalloidin-AlexaFluor^®^555. Coverslips were mounted by inverting them on glass slides using Prolong anti-fade mounting media (Molecular Probes). Coverslips were examined on a Zeiss Axiovert 200 M microscope (Zeiss) using 40X or oil immersion 63× objective lens. Fluorescent images were captured using a CoolSnap™ES camera (Photometric^®^, Roper Scientific) and analyzed using Metamorph^® ^(V6.3, Molecular Devices Corp.).

### Tumor growth in vivo

WM278 primary melanoma cells either parental, overexpressing GFP (C2) or GFP-Nck2 at low (N7) or high (N14) levels were grown in RPMI medium supplemented with 10% FBS to 80% confluency. Cells (5 × 10^6^) resuspended in 500 μl at 50% Matrigel™ were injected subcutaneously in the right flank of 6-week-old CD-1 Nude mice (Charles River, Qc, Canada) (n = 5 for each group). Tumors development was followed for 20 weeks. Tumor size was measured every week with calipers to assess tumor volume ([πlength × width^2^]/6). Mice were housed in McGill University Animal facilities at the Genome building. Mice experiments were conducted under a McGill University-approved animal use protocol (Dr. P.M. Siegel) in accordance with guidelines established by the Canadian Council on Animal Care.

### Data analysis and statistics

Densitometry analysis results are expressed as means ± S.E.M. Student's *t *test was used to evaluate the statistical significance of the results. A p ≤value 0.05 is assumed to be significant.

## Results

### Nck2 protein and mRNA levels are increased in human metastatic melanoma cell lines

To investigate the potential involvement of Nck proteins in human melanoma development and progression, we first analyzed total Nck protein levels in human melanoma cell lines at different stages of cancer progression and compared with normal human melanocytes. The human melanoma cell lines used in this study were provided by the laboratory of Dr. Meenhard Herlyn at the Wistar Institute (PA, USA) and already used *in vivo *for tumorigenicity and experimental metastasis [23,24]. Mainly, these include the WM278, a melanoma cell line derived from a human primary tumor in vertical growth phase that rarely metastasis; WM1617, a WM278 sister melanoma cell line derived from lymph nodes metastasis in the same patient few years later; 451Lu, a melanoma cell line isolated from lung metastasis in mice injected with the WM164 cell line, which is a human melanoma cell line isolated from lymph nodes metastasis similar to WM1617, but from a different patient.

**Figure 1:**
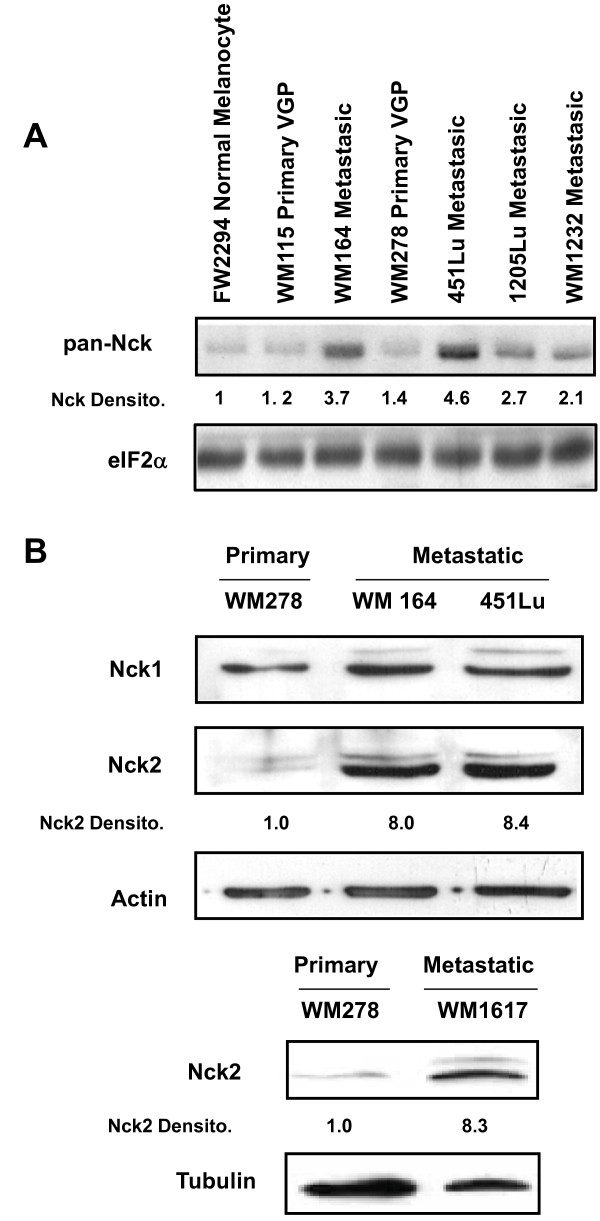
**Nck expression in human melanoma cell lines at different stages of cancer progression**. Equivalent amount of total proteins from human primary and metastatic melanoma cell lysates (30 μg) were subjected to western blot analysis using indicated antibodies. (A) Pan-Nck antibodies recognize Nck1 and Nck2. eIF2α was used as loading control. (B) Nck1 and Nck2 are specific Nck isotypes antibodies (Additional file 1) and β-actin or tubulin was detected as loading controls. Quantification of Nck1 and Nck2 signals, evaluated in the linear range of detection by densitometry, are reported below the blots. Human Melanoma Cell Lines Characteristics: FW2294: normal melanocyte; WM115: poorly metastatic primary melanoma in vertical growth phase and with round morphology; WM164: elongated metastatic melanoma isolated from lymph nodes; WM278: poorly metastatic primary melanoma in vertical growth phase and with mixed morphology; WM1617: elongated metastatic melanoma derived from lymph nodes, sister match of WM278; 451Lu: elongated metastatic melanoma selected in lungs of mice injected with WM164 cells; 1205Lu: round metastatic melanoma selected in mice; WM1232: round metastatic melanoma.

From western blots performed using a rabbit polyclonal antibody that equally recognizes both Nck isotypes (Pan-Nck, Additional file 1) [21], we observed higher levels of Nck proteins in highly metastatic melanoma (WM164, 451Lu, 1205Lu and WM1232) compared with weakly metastatic primary melanoma (WM115 and WM278) and normal melanocyte (FW2294) cell lines (Figure 1A). Further analyses using in-house generated Nck isoforms specific antibodies (Additional file 1) [15] revealed that increased expression of Nck in metastatic melanoma cells is mainly due to drastic higher expression levels of Nck2 (Figure 1B, compare WM164 and 451Lu with WM278). Additional comparison of highly metastatic (WM1617) and weakly metastatic (WM278) human melanoma cell lines isolated from the same patient (Figure 1B), further confirmed increased expression of Nck2 in human metastatic melanoma. Interestingly, Nck1 protein levels normalized according to actin or tubulin loading control were comparable among the human melanoma cell lines investigated (Figure 1B and Additional file 2). In addition, we also found no change in expression levels of other SH2/SH3 domain-containing signaling proteins, such as PLC-γ1, p85 of PI3K, Grb2 and Crk, as normalized for actin or tubulin loading control (Additional file 2 and data not shown). Altogether, these results suggest a specific role for Nck2 in human melanoma progression.

**Figure 2:**
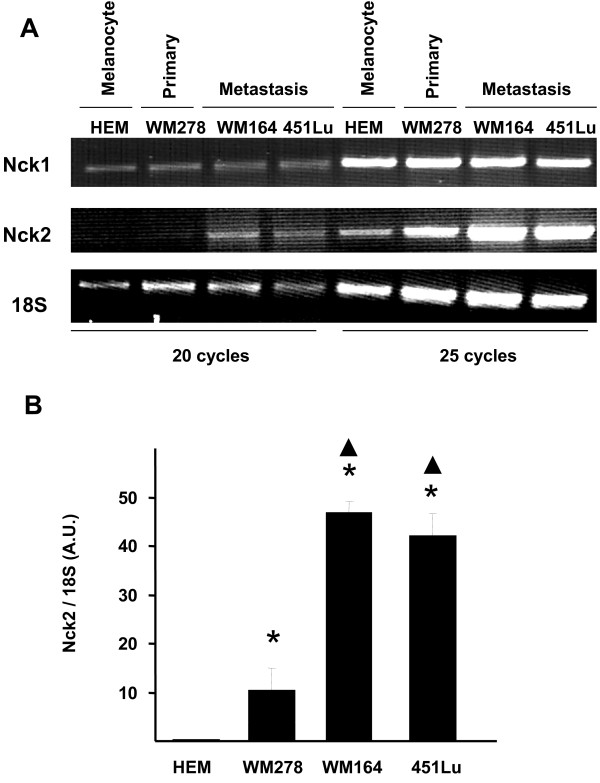
**Nck1 and Nck2 mRNA expression in human melanocytes and melanoma cells**. (A) RT-PCR amplification performed on isolated RNA from human melanocyte and melanoma cells using specific primers for human Nck1, Nck2 and 18S. Shown are PCR products after 20 and 25 cycles of amplification. HEM: human normal melanocytes; WM278: human primary melanoma that rarely metastasis; WM164: human melanoma isolated from metastasis in lymph nodes; 451Lu: lung melanoma metastasis of WM164 injected in mice. 18S is used as control. (B) Ratios of Nck1 and Nck2 mRNA over 18S were calculated from quantification by densitometry of PCR amplification products in the linear range. Asterisk: p ≤ 0.001 compared to HEM, black triangle: p ≤ 0.001 compared to WM278.

To assess whether increased expression of Nck2 protein levels in human metastatic melanoma cells correlates with upregulated transcription of *Nck2 *gene, we compared Nck1 and Nck2 mRNA levels in three human melanoma cell lines at different stages of progression and in human primary melanocytes (HEM) by performing RT-PCR using Nck isoforms specific primers. In the linear range of PCR amplification, no significant change in Nck1 mRNA levels was detected in all human melanoma cell lines and compared with HEM (Figure 2A). In contrast, we observed a strong increase in Nck2 mRNA levels in all human melanoma cell lines compared with HEM. In addition, compared with primary melanoma cells (WM278), metastatic melanoma cell lines (WM164 and 451LU) showed significant increased Nck2 mRNA levels (Figure 2B). Altogether, our results reveal that Nck2 protein and mRNA levels are significantly increased in different human metastatic melanoma cells compared with human weakly metastatic primary melanoma and melanocyte cells, suggesting Nck2 as a biological marker of human melanoma metastasis that could contribute to melanoma progression.

### Nck2 promotes human melanoma cell proliferation

**Figure 3:**
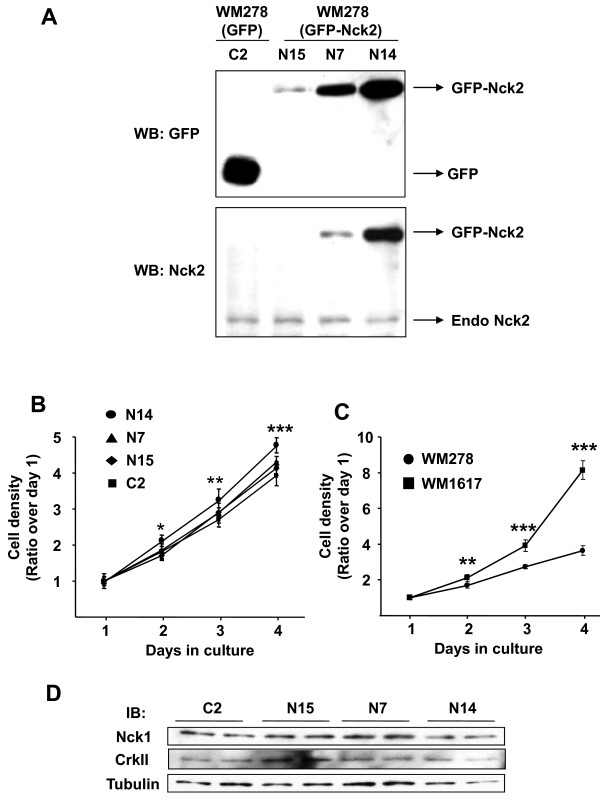
**Effect of Nck2 on human primary melanoma cell proliferation**. (A) Total cell lysates from stable WM278 human primary melanoma cells overexpressing GFP (C2) or increasing levels of GFP-Nck2 (N15 < N7 < N14) were subjected to western blot analysis using GFP or Nck2 specific antibodies. Cell proliferation of (B) WM278 cells stably overexpressing GFP (C2) or GFP-Nck2 (N15, N7 and N14), as well as (C) parental primary (WM278) and metastatic (WM1617) melanoma cells was determined by quantification of Crystal Violet incorporation each day during 4 days in culture. Results are shown as the ratio of the mean of cell density over day 1 ± SD (n = 4). * p < 0.02, ** p < 0.006 and ***p ≤ 0.0001compared with C2 (B) or parental WM278 cells (C) using Student's *t*-test. (D) Nck1 and CrkII expression was evaluated using total cell lysates (30 μg protein) prepared from WM278 cells stably overexpressing GFP (C2) or GFP-Nck2 (N15, N7, and N14). β-tubulin was used as loading control.

To demonstrate a role for Nck2 in melanoma progression, we first determined whether Nck2 regulates cell proliferation in WM278 human primary melanoma cells, which endogenously express low levels of Nck2 protein and rarely metastasis compared with its metastatic counterpart WM1617 melanoma cells. For this, we created WM278 cell lines stably overexpressing increasing levels of GFP-Nck2 (N15 < N7 < N14) or GFP as control (C2) (Figure 3A). Using these WM278 stable cell lines, we found that overexpressing high levels of Nck2 significantly enhanced cell proliferation (Figure 3B, left panel, compare clone N14 with C2). It is interesting to note though that the effect of Nck2 on WM278 primary melanoma cells proliferation seems to parallel the levels of Nck2 overexpressed (Figure 3A and 3B, compared N15, N7 and N14). In agreement, the WM1617 human metastatic melanoma cells that endogenously express higher levels of Nck2 compared with human primary melanoma cells, also show higher proliferative abilities than its paired WM278 primary melanoma cells that rarely metastasis (Figure 3C). As expected, we found no change in the protein levels of Nck1 or CrkII, a SH2-SH3 domain-containing adaptor protein previously identify as an oncogene [25] and recently reported to regulate sarcoma cell proliferation [26] (Figure 3C).

**Figure 4:**
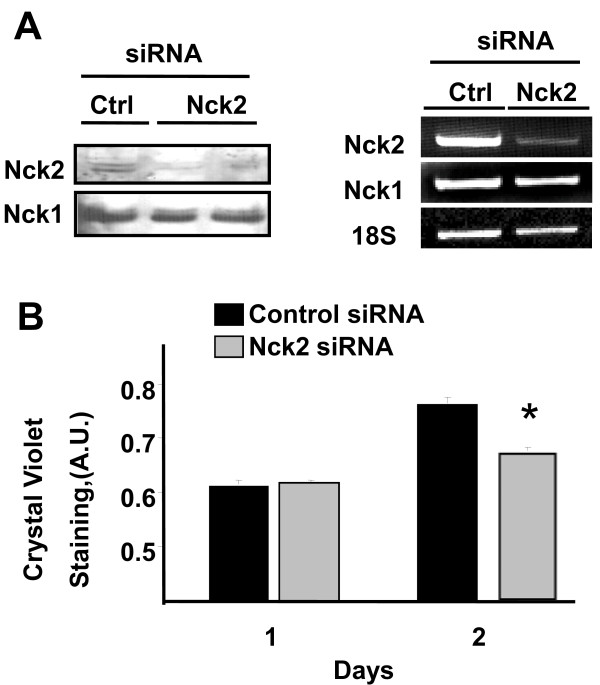
**Effect of Nck2 on human metastatic melanoma cell proliferation**. (A) Total cell lysates (30 μg protein) prepared from 451Lu human melanoma cells transfected with control or specific Nck2 siRNA were subjected to western blot analysis using specific antibodies that recognize Nck1 or Nck2 (left panel). RT-PCR was performed to determine Nck1 and Nck2 mRNA levels using total RNA and specific primers for human Nck1 and Nck2. 18S was used as control (right panel). (B) 451Lu human melanoma cells transfected with control or Nck2 siRNA were followed for proliferation 1 and 2 days after siRNA transfection. Results represent cell density corresponding to the mean of Crystal Violet incorporation ± SD from one experiment performed in triplicate. * p < 0.05 vs control siRNA using Student's *t*-test. Similar results were found in three independent experiments.

To confirm a role for Nck2 in melanoma cell proliferation, we assessed whether siRNA-mediated downregulation of Nck2 in human metastatic melanoma cells affects cell proliferation. As shown in Figure 4A, Nck2 siRNA transfection of 451Lu metastatic melanoma cells resulted in decreased Nck2 protein and mRNA levels, while Nck1 protein and mRNA levels were not altered. More importantly, we found that cell proliferation was significantly decreased in Nck2 depleted metastatic melanoma cells compared with control siRNA treated cells (Figure 4B). To rule out that this effect was due to increased cell death in Nck2 depleted melanoma cells, 2 days post transfection we evaluated the percentage of cells with nuclei stained by Trypan Blue and observed no difference between metastatic melanoma cells transfected with control (8.7% ± 2.5) and Nck2 (7.7% ± 1.7) siRNA. Altogether, these results indicate that Nck2 contributes to the control of proliferation in human melanoma cells.

### Nck2 modulates migration and invasion of human melanoma cells

**Figure 5:**
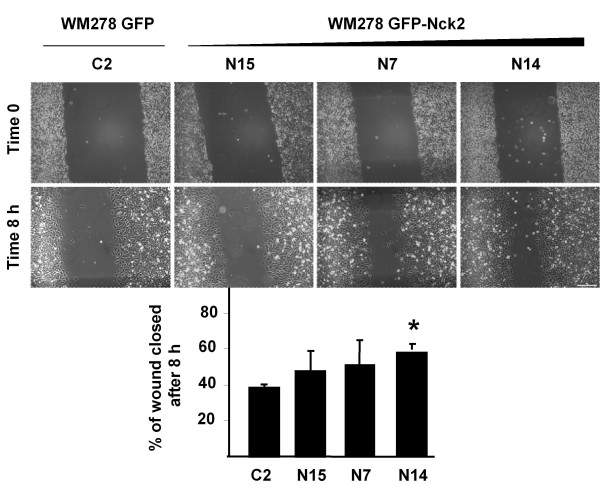
**Effect of Nck2 on human primary melanoma cell migration**. Human primary melanoma cell migration was evaluated using wound healing assays on WM278 cells overexpressing GFP (C2) or increasing levels of GFP-Nck2 (N15 < N7 < N14). Pictures from the same area were taken at time 0 and 8 hours after the wound. Magnification: 10X and white bar = 200 μm. Quantification of migration is expressed as percentage of closed wound ± SD. * p < 0.001 vs C2 using Student's *t*-test.

Cancer progression involves that transformed cells must acquire motility and invasive activities. Therefore, we next determined whether Nck2 was critical to melanoma cell migration and invasion. We compared migration of WM278 primary melanoma cells overexpressing GFP (C2) or increasing levels of GFP-Nck2 (N15 < N7 < N14) in wound healing assays. As shown in Figure 5, increasing levels of GFP-Nck2 in WM278 melanoma cells promoted migration and this was significant in cell line N14, which expresses higher levels of Nck2 proteins compared with N5 and N7 cell lines (Figure 3A). To exclude that a clonal effect is responsible of increased migration of WM278 melanoma cells overexpressing GFP-Nck2, we transiently overexpressed HA-Nck2 in WM278 primary melanoma cells using retroviral infection (Additional file 3). In this context, we still observed a significant increase in migration of WM278 human primary melanoma cells overexpressing HA-Nck2 compared to control infected WM278 melanoma cells in wound healing assays. It is interesting to note though that the effect of Nck2 on migration was already observed 8 hours after wounding, suggesting that cell proliferation is not involved.

**Figure 6:**
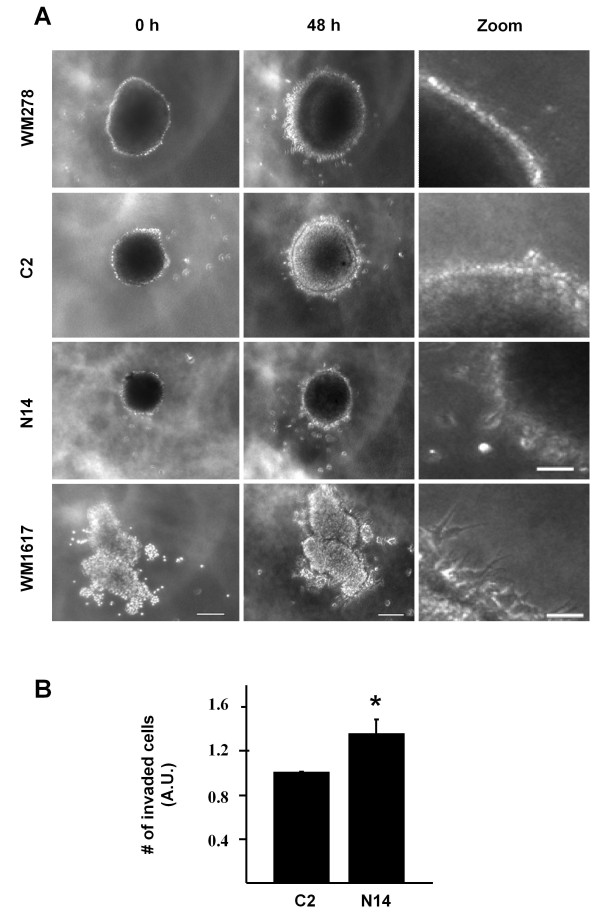
**Effect of Nck2 on human primary melanoma cell derived spheroid in type 1 collagen and invasion**. (A) Spheroids of human primary WM278 melanoma cells overexpressing GFP (C2) or GFP-Nck2 (N14) and human metastatic WM1617 melanoma cells embedded in 3D type I collagen gel were analyzed for morphology and invading cells. Pictures (10X) were taken at time 0 and after 48 hours of culture. White bars = 200 μm or 50 μm for zoomed pictures. (B) Invasion of human primary WM278 melanoma cells overexpressing GFP (C2) or GFP-Nck2 (N14) was evaluated using Matrigel™ transwell assays. Results are expressed as the mean of invaded cells ± SD from one experiment performed in triplicate. * p < 0.01 vs C2 using Student's *t*-test. Similar results were observed in three independent experiments.

To determine whether overexpression of Nck2 in primary melanoma cells promotes invasion in a tumor-like context, we evaluated migration of melanoma cells at the edge of multicellular spheroids embedded into a collagen type I matrix (Figure 6A). As expected, spheroids of human primary melanoma cells overexpressing or not GFP (WM278, C2) grew as compact units devoid of cells migrating outward after 48 h in culture. In contrast, WM1617 human metastatic melanoma cells after 48 h in culture formed open-wave fragile spheroids with cells in periphery sending long projections invading the surrounding collagen. These observations are in agreement with the established invasive phenotype of WM1617 melanoma cells. Interestingly, WM278 human primary melanoma cells overexpressing GFP-Nck2 (N14) apparently did not show similar extensions from spheroid border cells, but form less compact spheroids than WM278-GFP with individual cells that detached form the mother spheroid after 48 h of culture into collagen gel. This observation reveals that overexpression of Nck2 in primary melanoma cells may contribute to invasiveness by promoting cell detachment and migration from primary melanoma lesion *in vivo*. In agreement, we found that Nck2 overexpression significantly promoted primary melanoma cells invasion through Matrigel™ matrix in transwells assays (Figure 6B). Altogether, these results suggest that Nck2 promotes cell migration and invasion in human melanoma cells.

### Nck2 modulates focal adhesions in human melanoma cells

**Figure 7:**
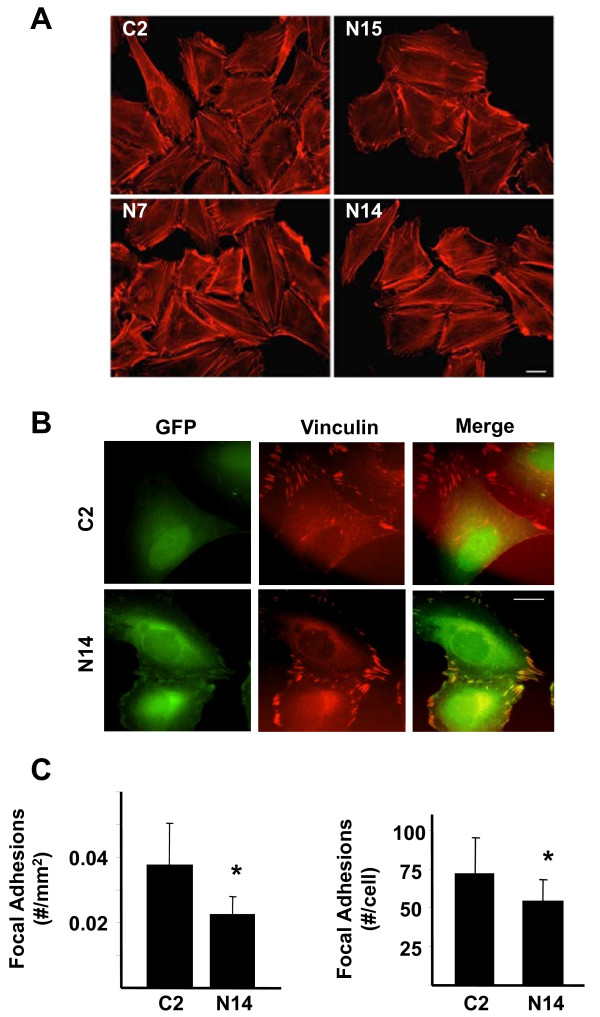
**Effect of Nck2 on actin organization and focal adhesions in human primary melanoma cells**. (A) WM278 human primary melanoma cells overexpressing GFP (C2) or increasing levels of GFP-Nck2 (N15 < N7 < N14) were submitted to actin staining using phalloidin-coupled to AlexaFluor^®^555. Pictures were taken at 40X and white bar represent 20 μm. (B) Fluorescence pictures of WM278 human primary melanoma cells stably overexpressing GFP (C2) or GFP-Nck2 (N14) subjected to vinculin staining using anti-vinculin specific antibody and GAM-TRICT (63X, white bar: 20 μm). (C) Manual quantification of focal adhesions as positive vinculin structures in 50 representative cell types. Results normalized for cell size are expressed as the mean of focal adhesion per mm^2 ^or cell ± SD. * p < 0.001 vs C2 using Student's *t*-test.

Because Nck adaptor proteins play an important role in regulating actin cytoskeleton reorganization, we then compared actin staining in WM278 human primary melanoma cells expressing either GFP (C2) or increasing levels of GFP-Nck2 (N15 < N7 < N14) (Figure 7A). Regardless of Nck2 expression levels, we found no apparent change in actin staining in these cells. This suggests that overexpression of Nck2 has no major effect on actin polymerization and organization, as well as on overall cell morphology in human primary melanoma. In contrast, vinculin staining, which shows that GFP-Nck2 colocalizes with vinculin at focal adhesions (Figure 7B), revealed significant reduced number of focal adhesions in human primary melanoma cells overexpressing Nck2 (N14) compared with control melanoma cells (C2) (Figure 7C). Therefore, these data suggest that increased expression of Nck2 in human primary melanoma cells might facilitate melanoma migration by decreasing focal adhesions.

### Nck2 promotes phosphorylation of proteins on tyrosine and downregulation of cell surface adhesion proteins in human primary melanoma cells

**Figure 8:**
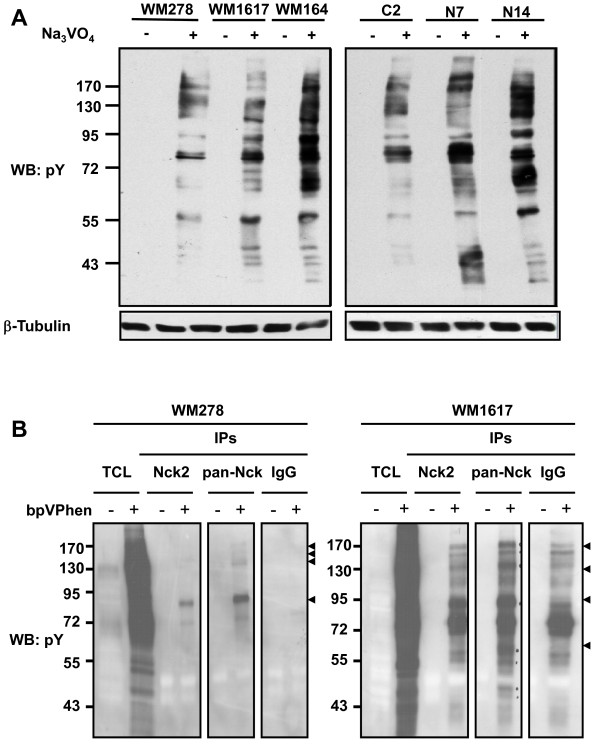
**Effect of Nck2 on phosphotyrosine proteins in human melanoma cells**. (A) Tyrosine phosporylated proteins in total cell lysates (30 μg protein) prepared from human primary (WM278) or metastatic (WM1617, WM164) melanoma cells, as well as from WM278 cells overexpressing GFP (C2) or GFP-Nck2 (N7, N14), treated with or without pervanadate (Na_3_VO_4 _at 100 μM, 15 min at 37°C), were evaluated by western blot using specific p-Tyr antibody. β-tubulin was probed as loading control. (B) Tyrosine phosporylated proteins coimmunoprecipitated with Nck2, pan-Nck or normal rabbit IgGs from human primary WM278 and metastatic WM1617 melanoma cell lysates were revealed by western blot using specific p-Tyr antibody. Arrow heads show proteins specific to or increased in Nck2 and pan-Nck IPs. Results shown are typical of 3-5 independent experiments.

**Figure 9:**
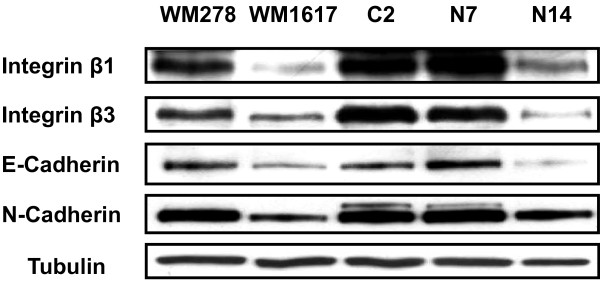
**Effect of Nck2 on cadherins and integrins expression in human melanoma cells**. Total cell lysates (50 μg protein) prepared human primary (WM278) or metastatic (WM1617) melanoma cells, as well as WM278 cells overexpressing GFP (C2) or GFP-Nck2 (N7, N14) were probed for cadherins and integrins using specific antibodies. β-tubulin used as loading control.

Protein tyrosine phosphorylation is a critical mechanism regulating focal adhesion dynamics [27,28]. Substantial evidence support a role for protein tyrosine kinases in focal adhesions assembly/disassembly toward the formation of invasive adhesion structures called invadopodia during cancer progression [29-33]. To investigate the mechanism by which Nck2 overexpression impinges on the phenotype of primary melanoma cells, we compared the levels of tyrosine phosphorylated proteins between human melanoma cells expressing different levels of Nck2 protein. To evaluate protein tyrosine phosphorylation, we exposed melanoma cells to pervanadate, a potent protein tyrosine phosphatase inhibitor [34-36] that allows tyrosine phosphorylated proteins to accumulate before harvesting the cells and performing immunoprecipitation. In these conditions, as observed in human metastatic (WM1617, WM164) compared with (WM278) melanoma cells (Figure 8A), WM278 cells overexpressing GFP-Nck2 presented increasing levels of proteins phosphorylated on tyrosine residues than WM278 control cells overexpressing GFP (Figure 8A). In addition, we found that tyrosine phosphorylated proteins coimmunoprecipitated with Nck2 (Nck2 IP) or total Nck (pan-Nck IP) were more abundant in human metastatic melanoma WM1617 cells compared with the counterpart WM278 primary melanoma cells (Figure 8B). We discovered also that like the metastatic WM1617 melanoma cells, the WM278 primary melanoma cells overexpressing high levels GFP-Nck2 (N14) displayed low levels of Integrin β1 and β3, as well as E- and N-Cadherin in Triton X-100 soluble extracts compared either with parental WM278 cells, WM278 cells overexpressing GFP (C2) or low levels of GFP-Nck2 (N7) (Figure 9). Collectively, these results reveal that increased expression of Nck2 in human primary melanoma cells promotes phosphorylation of proteins on tyrosine, concomitant with the assembly of Nck2-dependent pY-proteins containing molecular complexes and downregulation of cell surface adhesion proteins.

### Nck2 promotes primary melanoma-derived tumor growth in vivo

**Figure 10:**
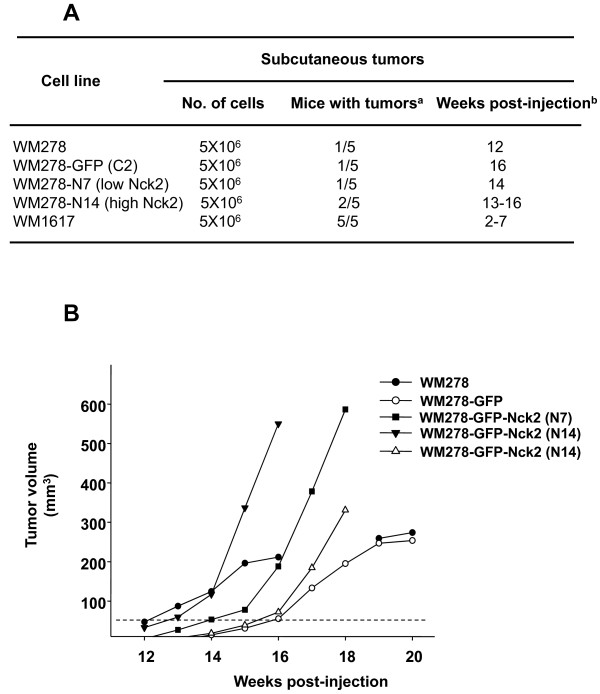
**Effect of Nck2 on human primary melanoma-derived tumor growth *in vivo***. CD-1 Nude mice of 6-week-old were injected subcutaneously with human parental WM278 primary melanoma cells, WM278 cells overexpressing either GFP or GFP-Nck2, or human WM1617 metastatic melanoma cells (5 × 10^6^) into the right flank. (A) The mice were monitored for tumor development. (B). Tumor volume progression assessed with calipers every week in indicated mice. Tumors with volume bigger than 50 mm^3 ^(dashed line) were considered during the course of the study.

To establish the biological relevance of our findings, we examined whether overexpression of Nck2 in human primary melanoma cells confers some tumorigenic advantage in a xenograft mouse model. To test this, WM278 human primary melanoma cells overexpressing GFP-Nck2 at low (N7) or high (N14) levels, along with parental WM278 cells, WM278 cells overexpressing GFP (C2) and WM1617 human metastatic melanoma cells were injected subcutaneously into CD-1 Nude mice. Two out of 5 mice injected with WM278 human primary melanoma cells overexpressing high levels of GFP-Nck2 (N14) developed tumor at the site of injection between 13-16 weeks post injection (Figure 10A). At the same time, 1 out of 5 mice that received either parental WM278 cells, WM278 melanoma cells overexpressing GFP (C2) or overexpressing low levels of GFP-Nck2 (N7) presented a subcutaneous tumor at the site of injection. In contrast, all mice injected with the metastatic WM1617 cells developed tumors at the site of injection 2-7 weeks post-injection (Figure 10A). As expected, tumors derived from metastatic WM1617 cells grew rapidly and reached maximal volume allowed between 5-11 weeks post-injection. Altogether, these observations suggest that increased expression of Nck2 in human melanoma cells is not sufficient to promote the appearance of subcutaneous tumor derived from melanoma. However, melanoma-derived tumor growth rate in mice injected with WM278 cells overexpressing GFP-Nck2 (N7 and N14) was greatly enhanced compared with tumor found in mice injected with WM278 cells parental or overexpressing GFP (C2) (Figure 10B), suggesting that increased expression level of Nck2 promotes primary melanoma-derived tumor growth rate. Subcutaneous tumors derived from WM278 cells overexpressing GFP-Nck2 (N7 and N14) could not be further monitored than few weeks after their occurrence due to the appearance of important spontaneous tumor necrosis core that required mice to be euthanized. Nevertheless, in line with our *in vitro *studies (Figure 3B), these results strongly support a role for Nck2 in melanoma-derived tumor growth rate *in vivo*.

### Nck2 expression is upregulated in invasive colon and breast cancer cell lines

**Figure 11:**
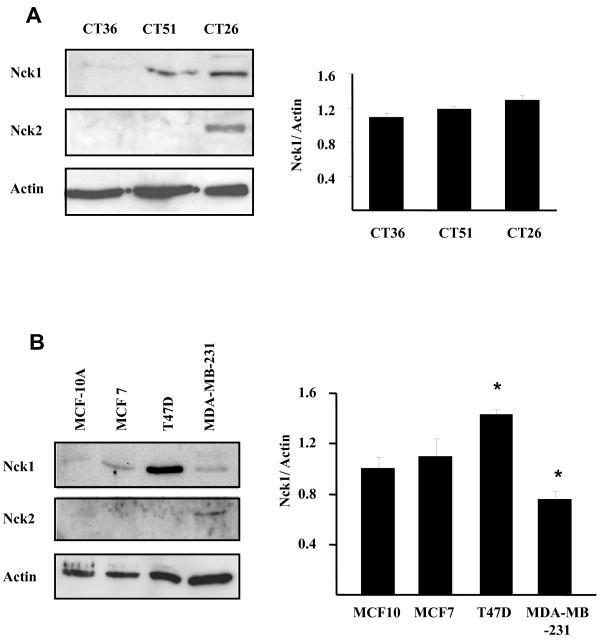
**Expression of Nck1 and Nck2 in colon and breast cancer cells**. Lysates (50 μg protein) from murine colonic carcinoma cells (A) and human breast cancer cells (B) were subjected to western blot analysis using anti-Nck specific antibodies (Additional file 1). CT36: rarely metastatic; CT51: intermediary; CT26: highly metastatic. MCF-10A: Normal human breast epithelial; MCF7, T47D, MDA-MB-231: invasive metastatic ductal carcinoma. For both cancer cell types, β-actin was probed as loading control.

To find out whether increased expression of Nck2 during cancer progression is observed in other cancer types than in melanoma skin cancer, we assessed Nck isoforms protein levels in murine colon (CT) and human breast cancer cell lines at different stages of progression (Figure 11). The CT represent mouse tumorigenic colonic carcinoma cell lines established in culture from three transplantable murine tumors of colonic origin at different stages of progression [37]. Based on growth rate and metastatic spreading, CT26 is the most aggressive, while CT51 is intermediary and CT36 is the least and rarely metastasizes. Using these cells lines, we observed that Nck1 protein levels are increased in the metastatic CT26 and CT51, while just barely detected in CT36. However, when Nck1 expression levels were normalized according to actin, these variations were not statistically significant. In contrast, Nck2 that was below detection level in CT36 and CT51, was distinctly detected in CT26, revealing increased expression of Nck2 in aggressive metastatic colon cancer cells. To determine whether expression of Nck isoforms vary during breast cancer progression, we selected few of the widely used human breast cancer cell lines (MCF-7, T-47D and MDA-MB-231) and an immortalized normal human breast epithelial cell line (MCF10A). MCF-7, T-47D and MDA-MB-231 are invasive ductal carcinoma of similar origin with metastatic properties [38]. Independently of estrogen receptor expression (ER+: MCF-7 and T-47D; ER-: MDA-MB-231) or epithelial/mesenchymal phenotype (epithelial-like low invasion: MCF-7 and T-47D; mesenchymal-like high invasion: MDA-MB-231), Nck1 protein levels in breast cancer cells were not consistent with the cancer stage. Considering actin as loading control, Nck1 protein levels were significantly increased in T-47D cells, decreased in MDA-MB-231 cells and not change in MCF7 cells compared to MCF-10A cells, excluding a potential correlation between Nck1 expression levels and breast cancer progression. In contrast, Nck2 was only detected in the most aggressive MDA-MB-231 cells, which are mesenchymal-like ER+ breast cancer cells with strong migratory and metastatic abilities [38,39]. Together these results provide the first evidence that the expression of Nck2 is increased in metastatic cancer cells of various origins and argue for a role of Nck2 in cancer progression.

## Discussion

Nck1 and Nck2 SH2-SH3 domain-containing proteins have been reported to be differently expressed in numerous mouse tissues [8,15]. In agreement with the ability of both Nck to collaborate with strong oncogenes to transform cells [6], *Nck1 *and *Nck2 *genes were found upregulated in several human cancer cell lines, including melanoma (https://www.oncomine.org/). However, Nck proteins expression levels in cancer tissues and possible mechanism(s) by which these adaptors contribute to cancer development have been poorly investigated to date. In this study, we provide evidence that Nck2 plays a role in promoting proliferation, migration and invasion of human melanoma cells *in vitro *and growth of melanoma-derived tumors *in vivo*, while its expression is upregulated in metastatic cancer cells, including colon, breast and melanoma.

Our investigation revealing that Nck2 overexpression in human primary melanoma cells induces metastatic characteristics point towards Nck2 sufficiency to promote metastasis phenotype. In this study, we did not address whether Nck2 is necessary for melanoma metastasis. However, we provided some insights suggesting that Nck2 could play such function. In fact, we found higher levels of Nck2 expression in metastatic compared to non metastatic cell lines in three different types of cancer. In addition, we demonstrate that depletion of Nck2 in metastatic melanoma reduces cell proliferation. This does not exclude that other yet identified players could be required to fully promote metastasis in melanoma overexpressing Nck2. None the less, our findings clearly demonstrate that overexpression of Nck2 in human primary melanoma correlates with upregulation of the total phospho-tyrosine proteins content, assembly of novel Nck2-dependent pY-protein complexes and downregulation of E- and N-cadherins, and β-1 and -3 integrins. E-cadherin, found at adherens junctions, is the principal effector of cell-cell adhesion [40]. Loss of E-cadherin expression in cancer cells weakens cell-cell adhesion and is associated with cancer progression, invasion and metastasis [41-43]. At the present time, there is no evidence for a direct link between E-cadherin and Nck2. Further investigation is required to elucidate the molecular events responsible for E-cadherin downregulation associated with overexpression of Nck2 in human primary melanoma cells and whether downregulation of Nck2 in metastatic human melanoma cells would restore E-cadherin expression remains to be determined. On the other hand, the degree of cancer cells cohesion in primary tumor also depends on the strength of cell-ECM contacts mediated by integrins [44]. Alteration in integrins expression has been also implicated in cancer progression, invasion and metastasis [45,46]. Integrins signaling associated with regulation of the actin cytoskeleton leading to adhesive attachment involves the activation of the focal adhesion kinase (FAK) and the integrin-like kinase (ILK) (reviewed in [47]). Interestingly, Nck2 has been shown to affect cell motility through its direct interaction with FAK [48]. Moreover, increasing evidence support a close relationship between integrins and growth factor receptor tyrosine kinases to activate signaling pathways that promote proliferation and metastatic activity (reviewed in [49]). Nck2 has been reported to function as a molecular link connecting integrins and growth factor receptor tyrosine kinases signaling pathways. In fact, Nck2 associates with numerous receptor tyrosine kinases [17,50-54] through its SH2 domain and using its third SH3 domain, it binds to a LIM domain in PINCH (Particularly Interesting Cys-His-rich Protein) [55]. PINCH, a binding protein for ILK, plays an important role in mediating integrins-induced cell-ECM interaction by directing ILK to focal adhesions [56]. It is recognized that the ILK-PINCH complex participates to signaling pathways regulating fundamental cellular processes (reviewed in [57], including cell shape and migration [58]. A crucial role for Nck in regulating these processes was particularly illustrated by the findings showing that fibroblasts derived from *Nck *double knockout mice embryos display major defects in cell attachment, cell motility and actin remodeling [8]. Therefore, increased expression of Nck2 in human primary melanoma cells may elicit protein interactions that re-wire signaling pathways in a fashion that alters focal adhesions and promotes cell motility by interacting with FAK and PINCH. Alternatively, increased expression of Nck2 could passively destroy proper stoichiometry of molecular complexes and in this manner, indirectly contributes to cancer progression by altering signaling pathways regulating the actin cytoskeleton network supporting cell migration.

Nck proteins are known to couple activated receptor tyrosine kinases, as well as non receptor tyrosine kinases, to effectors involved in signaling pathways regulating proliferation and actin cytoskeleton dynamics [7,14,59-61]. Non-receptor protein kinases of the Src and Abl families are often overexpressed or aberrantly activated in a wide variety of human cancers and their roles in cancer progression, including proliferation, survival, motility, invasiveness, metastasis and angiogenesis, is significant. Of note, Nck directly binds to and promotes Abl activation and signaling [62,63], and associates with p60^v-src ^*in vitro *[16]. c-Src has been recently reported to be overexpressed in human metastatic melanoma tumors [64]. Interestingly, Src-dependent phosphorylation of Tks5 and cortactin recruits Nck to invadopodia, where it regulates actin assembly and ECM degradation [65-67]. Invadopodia, exclusive invasive cancer cell membrane actin-based protrusions enriched in signaling and proteolytic activities, are used by invasive cancer cells to degrade the ECM and invade surrounding tissues [68-71]. It is then possible that upregulation of tyrosine phosphorylated proteins and downregulation of cadherins and integrins in human primary melanoma cells that overexpress Nck2 may endow melanoma cells with altered adhesive properties and spatial relationships that favor uncontrolled proliferation, migration and invasion.

Nck1 and Nck2 proteins are highly identical, but despite high homology, redundant functions and common binding partners, increasing evidence suggest specific roles and protein interactions [7,9-12,14], as well as specific tissue expression patterns for Nck proteins [8,15]. In this study, the effect of Nck1 overexpression on melanoma phenotype was not addressed. However, our results demonstrate that increased endogenous expression of Nck2 in human metastatic melanoma cells relative to primary melanoma cells and melanocytes results from increased *Nck2 *transcription, suggesting that Nck1 and Nck2 promoters are under different regulatory controls.

## Conclusions

In conclusion, in this study we provide evidence for a role of the adaptor protein Nck2 in melanoma proliferation, migration and invasion *in vitro *and melanoma-derived tumor growth *in vivo*. Collectively, our data support Nck2 as a cornerstone governing the aspects that promote melanoma progression. Given other common metastatic cancer cell lines also overexpress Nck2, a general paradigm could make Nck2 a potential molecular marker of cancer progression and a novel target for anti-cancer drug therapy.

## Competing interests

The authors declare that they have no competing interests.

## Authors' contributions

MLC participated to most experiments and contributed to the analysis of the data. JD performed experiments to generate Figure 8 and has been essential to the study design and the draft the manuscript. SI participated in the cellular and biochemical assays. APC carried out wound healing assays experiments reported in Additional file 3. PMS contributed to the conception and achievement of *in vivo *experiments. LL conceived the study, participated in its design and coordination, and to the preparation of the final manuscript. All authors read and approved the final manuscript.

## Pre-publication history

The pre-publication history for this paper can be accessed here:

http://www.biomedcentral.com/1471-2407/11/443/prepub

## Supplementary Material

Additional file 1 **Nck isoforms specific antibodies**. Equivalent amount of total cell lysates (25 μg protein) of 293HEK cells overexpressing similar levels of human HA-Nck1 or HA-Nck2 proteins were probed by western blots using indicated antibodies. Click here for file

Additional file 2 **Nck1 and CrkII expression in human melanoma cell lines at different stages of cancer progression**. Equivalent amount of total cell lysate proteins (30 ug) from various human melanoma cell lines were subjected to western blot analysis using anti-CrkII and anti-Nck1 specific antibodies. β-tubulin was probed as loading control. Click here for file

Additional file 3 **Effect of Nck2 on human primary melanoma cell migration**. Melanoma migration was evaluated 8 h post wounding in wound healing assays using WM278 human primary melanoma cells 24 h following infection with retrovirus transducing or not HA-Nck2. Quantification of melanoma migration is expressed as mean of wound closed in mm ± SEM. * p < 0.05 compared to control using Student's *t*-test. Click here for file
