# Supplementary material for: Nck2 promotes human melanoma cell proliferation, migration and invasion in vitro and primary melanoma-derived tumor growth in vivo
Source: BMC Cancer. 2011 Oct 12;11:443. doi: 10.1186/1471-2407-11-443 (PMC3198724; doi:10.1186/1471-2407-11-443)
Supplement: Additional file 1 — Nck isoforms specific antibodies. Equivalent amount of total cell lysates (25 μg protein) of 293HEK cells overexpressing similar levels of human HA-Nck1 or HA-Nck2 proteins were probed by western blots using indicated antibodies. [file 1471-2407-11-443-S1.PPT]

## Slide 1
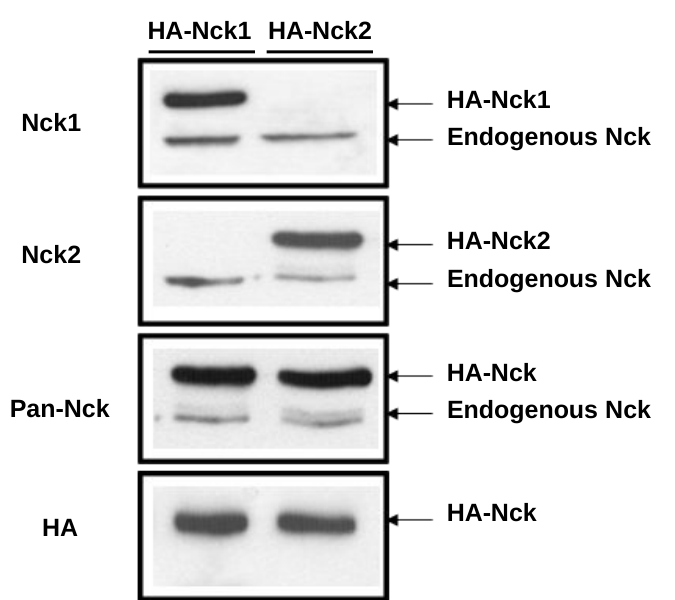

HA-Nck2
HA-Nck1
HA-Nck1
Nck1
Endogenous Nck
HA-Nck2
Nck2
Endogenous Nck
HA-Nck
Pan-Nck
Endogenous Nck
HA-Nck
HA
