# Supplementary figures and images for: Nck2 promotes human melanoma cell proliferation, migration and invasion in vitro and primary melanoma-derived tumor growth in vivo
Source: BMC Cancer. 2011 Oct 12;11:443. doi: 10.1186/1471-2407-11-443 (PMC3198724; doi:10.1186/1471-2407-11-443)

## Slide 1
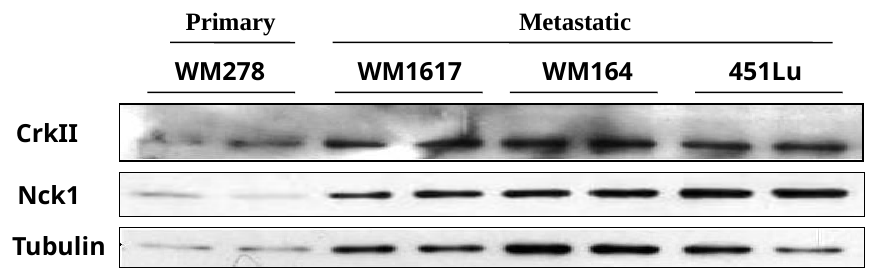

Primary Metastatic
WM278
WM1617
WM164
451Lu
CrkII
Nck1
Tubulin

Supplement: Additional file 2 — Nck1 and CrkII expression in human melanoma cell lines at different stages of cancer progression. Equivalent amount of total cell lysate proteins (30 ug) from various human melanoma cell lines were subjected to western blot analysis using anti-CrkII and anti-Nck1 specific antibodies. β-tubulin was probed as loading control. [file 1471-2407-11-443-S2.PPT]

## Slide 1
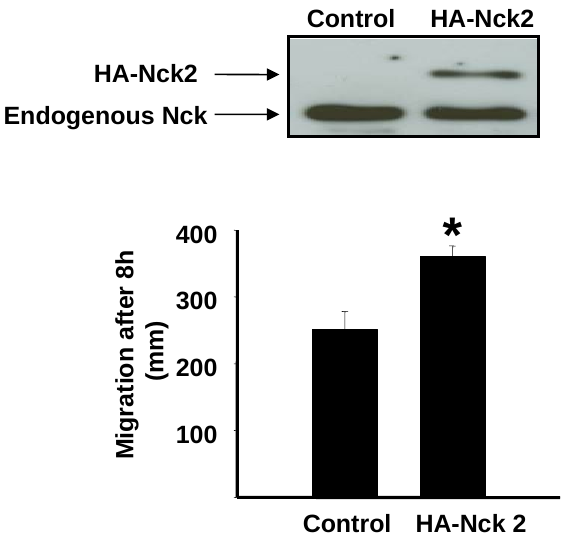

Control HA-Nck2
HA-Nck2
Endogenous Nck
*
400
300
Migration after 8h
 (mm)
200
100
Control
HA-Nck 2

Supplement: Additional file 3 — Effect of Nck2 on human primary melanoma cell migration. Melanoma migration was evaluated 8 h post wounding in wound healing assays using WM278 human primary melanoma cells 24 h following infection with retrovirus transducing or not HA-Nck2. Quantification of melanoma migration is expressed as mean of wound closed in mm ± SEM. * p < 0.05 compared to control using Student's t-test. [file 1471-2407-11-443-S3.PPT]
